# Supplementary material for: Two B-Box Proteins, MaBBX20 and MaBBX51, Coordinate Light-Induced Anthocyanin Biosynthesis in Grape Hyacinth
Source: Int J Mol Sci. 2022 May 19;23(10):5678. doi: 10.3390/ijms23105678 (PMC9146254; doi:10.3390/ijms23105678)
Supplement: Supplementary file 1 [file ijms-23-05678-s001.zip › ijms-1714932-supplementary.pdf]

Supplementary Material

Supplementary Figure

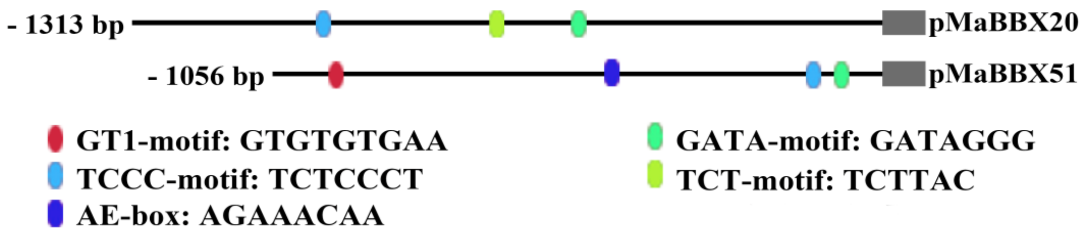

Figure S1. The light-responsive elements of the *MaBBX20* and *MaBBX51* promoters.

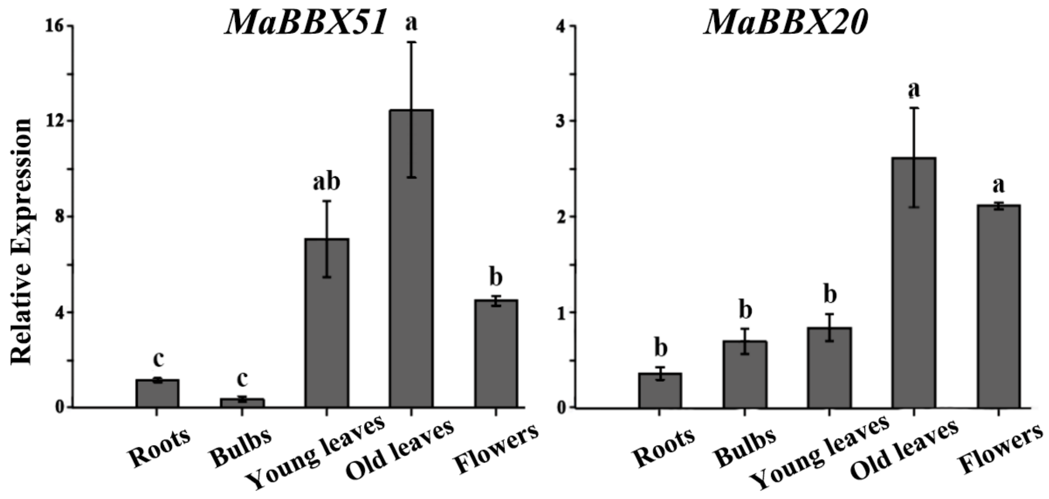

Figure S2. The expression patterns of MaBBX20 and MaBBX51 in various tissues (roots, bulbs, young leaves, old leaves, and flowers) of M. aucheri ‘Dark Eyes’. MaActin was used as the reference gene. Data are presented as the mean ± SD. Different letters above the bars indicate significantly different values calculated using one-way ANOVA followed by LSD analysis ($P < 0.05$).

Supplementary Tables

Table S1. Primer sequences.

**Primers for cloning**	**Sequence (5′ to 3′)**
MaBBX51-cDNA-full length-F	ATGAAGATTCAGTGTGATGCTTGTGAG
MaBBX51-cDNA-full length-R	CTATCCTAAATCTGGGACTGTGAAGTA
MaBBX20-cDNA-full length-F	GAGATTCCAGAACATCGGTTGCTACTG
MaBBX20-cDNA-full length-R	TGATGGTCATAGGAGGATGCATGTC
pMaBBX51-SP1	GGCATGAAGGATGATGGGCTTCCT
pMaBBX51-SP2	ATTGAACCTAGCGCCACACGGATACC
pMaBBX51-SP3	TGAAGGCAGGCTTTTCTTGGCAGAC
pMaBBX20-SP1	AGCTCCCCAGCAAGTACTTACACTGT
pMaBBX20-SP2	CCAGAGTAGCAGTTTATCGTATGCC
pMaBBX20-SP3	GCAGGCTGTGAAACACATCGATCGCA

**Primers for vector construction**	**Sequence (5′ to 3′)**
MaBBX51-2300-F- BamHI	CGAGCTCGGTACCCGGGGATCCATGAAGATTCAGTGTGATGCTTGTGAG
MaBBX51-2300-R- SalI	CTTGCTCACCATGGTGTCGACTCCTAAATCTGGGACTGTGAAGTA
MaBBX20-2300-F-BamHI	CGAGCTCGGTACCCGGGGATCCATGAAGATTCAGTGTGATGCTTGTGAG
MaBBX20-2300-R-SalI	CGAGCTCGGTACCCGGGGATCCTCCTAAATCTGGGACTGTGAAGTA
MaBBX51-GBKT7-F-SmaI	CATGGAGGCCGAATTCCCGGGATGAAGATTCAGTGTGATGCTTGTGAG
MaBBX51-GBKT7-R-SalI	ATGCGGCCGCTGCAGGTCGACCTATCCTAAATCTGGGACTGTGAAGTA
MaBBX20-GBKT7-F-SmaI	CATGGAGGCCGAATTCCCGGGATGGGCTCTCTTTGTGACTTCTGCGGG
MaBBX20-GBKT7-R-SalI	ATGCGGCCGCTGCAGGTCGACTCAGTAGCTTCGCGTC
MaBBX51-NE-F-BamHI	CCAGGCCTACTAGTGGATCCATGAAGATTCAGTGTGA
MaBBX51-NE-R-SalI	GCGGTACCCTCGAGGTCGACCTATCCTAAATCTGG
MaBBX20-NE-F-BamHI	CCAGGCCTACTAGTGGATCCATGGGCTCTCTTTGTGACTTCTG
MaBBX20-NE-R-SalI	GCGGTACCCTCGAGGTCGACTCCTAAATCTGGGACTG
MaHY5-NE-F-BamHI	CCAGGCCTACTAGTGGATCCATGCAGGAGCCGGCGACGAGCTC
MaHY5-NE-R-SalI	GCGGTACCCTCGAGGTCGACCTATGGCCCTTCACCAT
MaMybA-NE-F-BamHI	CCAGGCCTACTAGTGGATCCATGGAACCCAAGCTCAAGTCATC
MaMybA-NE-R-SalI	GCGGTACCCTCGAGGTCGACTCATAACAAGTTATGGGCATGGAGC

MaBBX51-CE-F-BamHI	GGCGCGCCACTAGTGGATCCATGAAGATTCAGTGTGA	
MaBBX51-CE-R-SalI	GCGGTACCCTCGAGGTCGACTCCTAAATCTGGGACTG	
MaBBX20-CE-F-BamHI	GGCGCGCCACTAGTGGATCCATGGGCTCTCTTTGTGACTTCTG	
MaBBX20-CE-R-SalI	TACATCCCGGGAGCGTCGACTCCTAAATCTGGGACTGTGAAG	
MaHY5-CE-F-BamHI	GGCGCGCCACTAGTGGATCCATGCAGGAGCCGGCGAC	
MaHY5-CE-R-SalI	TACATCCCGGGAGCGTCGACCTATGGCCCTTCACCATTGGCGCTAC	
MaBBX51-62sk-F-EcoRI	CCCCCGGGCTGCAGGAATTCATGAAGATTCAGTGTGATGCTTGTGAG	
MaBBX51-62sk-R-KpnI	GATTTCAGCGAATTGGTACCCTATCCTAAATCTGGGACTGTGAAG	
MaBBX20-62sk-F-EcoRI	CCCCCGGGCTGCAGGAATTCATGGGCTCTCTTTGTGACTTCTG	
MaBBX20-62sk-R-KpnI	GATTTCAGCGAATTGGTACCTCAGTAGCTTCGCGTCCGGCAGAG	
MaHY5-62sk-F-EcoRI	CCCCCGGGCTGCAGGAATTCATGCAGGAGCCGGCGACGA	
MaHY5-62sk-R-KpnI	GATTTCAGCGAATTGGTACCCTATGGCCCTTCACCATTGGCGCTACCGC	
MaMybA -62sk-F-EcoRI	CCCCCGGGCTGCAGGAATTCATGGAACCCAAGCTCAAGTCATC	
MaMybA -62sk-R-KpnI	GATTTCAGCGAATTGGTACCTCATAACAAGTTATGGGCATGGAGC	
pMaMybA-0800LUC-F-SalI	GGCCCCCCCTCGAGGTCGACAACGGCACCATAGCACGGTACAAT	
pMaMybA-0800LUC-R-BamHI	GCTCTAGAACTAGTGGATCCTATTAGCTTTTGCAGCAAGCTGAATTC	
pMaDFR-0800LUC-F-SalI	GGCCCCCCCTCGAGGTCGACGTGGAGTAGCATAGGAACGAGAG	
pMaDFR-0800LUC-R-BamHI	GCTCTAGAACTAGTGGATCCCTTGTTTGTGTGATTGTGTTGGGAG	
MaBBX51-GADT7-F-EcoRI	GCCATGGAGGCCAGTGAATTCATGAAGATTCAGTGTGATGCTTGTGAGAAGGCG	
MaBBX51-GADT7-R-BamHI	CTGCAGCTCGAGCTCGATGGATCCTCCTAAATCTGGGACTGTGAAGTACTCTTC	
MaBBX20-GADT7-F-EcoRI	GCCATGGAGGCCAGTGAATTCATGGGCTCTCTTTGTGACTTCTGCGGGGAGC	
MaBBX20-GADT7-R-BamHI	CTGCAGCTCGAGCTCGATGGATCCGTAGCTTCGCGTCCGGCAGAGTGGATCAT	
MaHY5-GADT7-F-EcoRI	GCCATGGAGGCCAGTGAATTCATGCAGGAGCCGGCGACGAGCTC	
MaHY5-GADT7-R-BamHI	CTGCAGCTCGAGCTCGATGGATCCTGGCCCTTCACCATTGGCGCTACC	
pMaMybA-AbAi-F-KpnI	TTCGAGCTCGGTACCAACGGCACCATAGCACGGTACAAT	
pMaMybA-AbAi-R-SalI	ATGCCTCGAGGTCGACTATTAGCTTTTGCAGCAAGCTGAATTC	
pMaDFR-AbAi-F-KpnI	TTCGAGCTCGGTACCGTGGAGTAGCATAGGAACGAGAG	
pMaDFR-AbAi-R-SalI	ATGCCTCGAGGTCGACCTTGTTTGTGTGATTGTGTTGGGAG	
**Primers for qPCR**	**Sequence (5′ to 3′)**	**Source**
MaBBX51-qPCR-F	GCCAAGAAAAGCCTGCCTTC	
MaBBX51-qPCR-R	GGATACCGGTTGCCAGGTAG	specifically designed
MaBBX20-qPCR-F	CCGGCAAGGAAAGCACATTC	

MaBBX20-qPCR-R	CGAATCTACCCTTCTCCCGC	
MaHY5-qPCR-F	TGGAGAGCGATGAGGAGATAA	Cao et al. 2019
MaHY5-qPCR-R	TAAGCCGCTTGTGCTCTTT	
MaDFR-qPCR-F	GAGAGAGCTGCATGGGATTT	Chen et al. 2017
MaDFR-qPCR-R	TGGCATGGTTGAGGTGATG	
MaANS-qPCR-F	GAGCCTCTCCCCTTCCAGCC	
MaANS-qPCR-R	TTCCACCTCATATTCCCCGC	
MaMybA-qPCR-F	TCCAGAGGTGTCGAAAGA	Chen et al. 2019
MaMybA-qPCR-R	TGTGTAGGCGCATGATAAG	
MaAN2-qPCR-F	CATGAACGGCAGACGAATCT	Chen et al. 2017
MaAN2-qPCR-R	GAAATTAGCATTCAAGCCATCCC	
MabHLH1-qPCR-F	GCAATGCTCAGTTTGCAGATAG	Chen et al. 2019
MabHLH1-qPCR-R	CTCTAGGACACCATCCATGAAG	
MaActin-qPCR-F	AACATTCAGAAAGAGTCCACCC	Chen et al. 2017
MaActin-qPCR-R	GCTTACCAGCAAAGATCAACCG	
NtCHS-qPCR-F	TGACACCCACTTGGATAGTTTAG	
NtCHS-qPCR-R	CGACCTCTGGAATTGGATCAG	
NtCHI-qPCR-F	CTTTTCTCGCCGCTAAATG	
NtCHI-qPCR-R	TTTCTGCCACCTTCTCTG	
NtF3H-qPCR-F	CAAGGCATGTGTGGATATGG	
NtF3H-qPCR-R	TGTGTCGTTTCAGTCCAAGG	
NtDFR-qPCR-F	AACCAACAGTCAGGGGAATG	
NtDFR-qPCR-R	TTGGACATCGACAGTTCCAG	
NtANS-qPCR-F	TGGCGTTGAAGCTCATACTG	
NtANS-qPCR-R	GGAATTAGGCACACACTTTGC	
NtUFGT-qPCR-F	GAGTGCATTGGATGCCTTTT	
NtUFGT-qPCR-R	CCAGCTCCATTAGGTCCTTG	
NtTubA1-qPCR-F	CTCCTATGCTCCTGTCATTTC	
NtTubA1-qPCR-R	GGCGAGGATCACACTTAAC	

Table S2. GenBank accession numbers for the known B-box proteins listed in Figure 1 and 2.

**Name**	**GenBank Accession Number**	**Species**
MaBBX51	MW160172	*Muscari aucheri* 'Dark Eyes'
MaBBX20	MW160173	*M. aucheri* 'Dark Eyes'
CmBBX8	KP963933	*Chrysanthemum indicum*
CmBBX13	KP963935	*Chr. indicum*
CmBBX24	KF385866	*Chr. indicum*
PpBBX24	Pdr4g016570	*Pyrus pyrifolia*
MdBBX1	MDP0000202669	*Malus domestica*
MdCOL4	MDP0000232445	*M. domestica*
AtBBX1	AT5G15840	*A. thaliana*
AtBBX2	AT5G15850	*A. thaliana*
AtBBX3	AT3G02380	*A. thaliana*
AtBBX4	AT2G24790	*A. thaliana*
AtBBX5	AT5G24930	*A. thaliana*
AtBBX6	AT5G57660	*A. thaliana*
AtBBX7	AT3G07650	*A. thaliana*
AtBBX8	AT5G48250	*A. thaliana*
AtBBX9	AT4G15250	*A. thaliana*
AtBBX10	AT3G21880	*A. thaliana*
AtBBX11	AT2G47890	*A. thaliana*
AtBBX12	AT2G33500	*A. thaliana*
AtBBX13	AT1G28050	*A. thaliana*
AtBBX14	AT1G68520	*A. thaliana*
AtBBX15	AT1G25440	*A. thaliana*
AtBBX16	AT1G73870	*A. thaliana*
AtBBX17	AT1G49130	*A. thaliana*
AtBBX18	AT2G21320	*A. thaliana*
AtBBX19	AT4G38960	*A. thaliana*
AtBBX20	AT4G39070	*A. thaliana*
AtBBX21	AT1G75540	*A. thaliana*
AtBBX22	AT1G78600	*A. thaliana*

AtBBX23	AT4G10240	*A. thaliana*
AtBBX24	AT1G06040	*A. thaliana*
AtBBX25	AT2G31380	*A. thaliana*
AtBBX26	AT1G60250	*A. thaliana*
AtBBX27	AT1G68190	*A. thaliana*
AtBBX28	AT4G27310	*A. thaliana*
AtBBX29	AT5G54470	*A. thaliana*
AtBBX30	AT4G15248	*A. thaliana*
AtBBX31	AT3G21890	*A. thaliana*
AtBBX32	AT3G21150	*A. thaliana*

Table S3. Transcription-related abbreviations and motifs in DNA

Abbreviations	Full Name
AE-box	AGAAACAA (5′ to 3′)
GATA motif	GATAGGG (5′ to 3′)
G-box	CACGTG (5′ to 3′)
GT1 motif	GTGTGTGAA (5′ to 3′)
TCCC motif	TCTCCCT (5′ to 3′)
TCT motif	TCTTAC (5′ to 3′)
